# Solventless synthesis of nanospinel Ni_1−*x*_Co_*x*_Fe_2_O_4_ (0 ≤ *x* ≤ 1) solid solutions for efficient electrochemical water splitting and supercapacitance†

**DOI:** 10.1039/d1ra04833c

**Published:** 2021-09-20

**Authors:** Nyemaga Masanje Malima, Malik Dilshad Khan, Jonghyun Choi, Ram K. Gupta, Philani Mashazi, Tebello Nyokong, Neerish Revaprasadu

**Affiliations:** Department of Chemistry, University of Zululand Private Bag X1001 KwaDlangezwa 3880 South Africa RevaprasaduN@unizulu.ac.za malikdilshad@hotmail.com; Department of Chemistry, College of Natural and Mathematical Sciences, University of Dodoma P. O. Box 338 Dodoma Tanzania; Institute of Physical Chemistry, Polish Academy of Sciences Kasprzaka 44/52 01-224 Warsaw Poland; Department of Chemistry, Pittsburg State University Pittsburg KS 66762 USA; Department of Chemistry, Rhodes University PO Box 94 Makhanda 6140 South Africa; Institute for Nanotechnology Innovation, Rhodes University PO Box 94 Makhanda 6140 South Africa

## Abstract

The formation of solid solutions represents a robust strategy for modulating the electronic properties and improving the electrochemical performance of spinel ferrites. However, solid solutions have been predominantly prepared *via* wet chemical routes, which involve the use of harmful and/or expensive chemicals. In the present study, a facile, inexpensive and environmentally benign solventless route is employed for the composition-controlled synthesis of nanoscopic Ni_1−*x*_Co_*x*_Fe_2_O_4_ (0 ≤ *x* ≤ 1) solid solutions. The physicochemical characterization of the samples was performed by p-XRD, SEM, EDX, XPS, TEM, HRTEM and UV-Vis techniques. A systematic investigation was also carried out to elucidate the electrochemical performance of the prepared nanospinels towards energy generation and storage. Based on the results of CV, GCD, and stability tests, the Ni_0.4_Co_0.6_Fe_2_O_4_ electrode showed the highest performance for the supercapacitor electrode exhibiting a specific capacitance of 237 F g^−1^, superior energy density of 10.3 W h kg^−1^ and a high power density with a peak value of 4208 W kg^−1^, and 100% of its charge storage capacity was retained after 4000 cycles with 97% coulombic efficiency. For HER, the Ni_0.6_Co_0.4_Fe_2_O_4_ and CoFe_2_O_4_ electrodes showed low overpotentials of 168 and 169 mV, respectively, indicating better catalytic activity. For OER, the Ni_0.8_Co_0.2_Fe_2_O_4_ electrode exhibited a lower overpotential of 320 mV at a current density of 10 mA cm^−2^, with a Tafel slope of 79 mV dec^−1^, demonstrating a fast and efficient process. These results indicated that nanospinel ferrite solid solutions could be employed as promising electrode materials for supercapacitor and water splitting applications.

## Abstract 2

The formation of solid solutions represents a robust strategy for modulating the electronic properties and improving the electrochemical performance of spinel ferrites.

## Graphical abstract

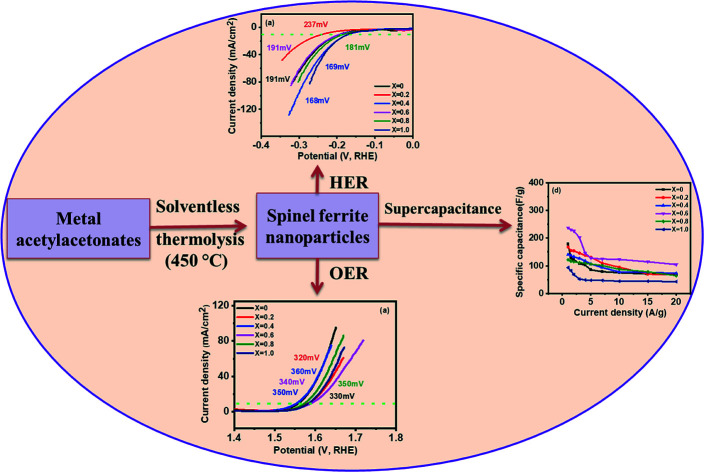

## 1. Introduction

In the wake of the increasing demand for clean and renewable energy supply coupled with rapid depletion and non-renewability of fossil fuels, innovative energy generation and storage technologies are highly desired.^1,2^ Electrocatalytic water splitting represents an efficient and flexible pathway for hydrogen production, a non-carbon-based alternative energy source. However, water splitting reactions have sluggish kinetics, large overpotential, and poor energy efficiency resulting from intricate processes of electrons/ions transfer in oxygen evolution (OER) and hydrogen evolution (HER) reactions.^3^ Although precious-metal-based catalysts have reportedly been employed to overcome these hurdles, their scarcity and substantial cost restrict their use in large-scale industrial applications.^4,5^ On the other hand, electrochemical capacitors are regarded as promising candidates for energy storage due to their long life cycle and high power density compared to rechargeable batteries.^6^ However, their relatively low energy density has prevented their widespread use in high-energy applications. In this regard, research endeavours are focused on developing earth-abundant, low-cost, and stable noble metal-free electrocatalysts for water splitting and supercapacitors.^7^

Spinel ferrites with a general formula AFe_2_O_4_ (where A is a divalent transition metal) are a fundamentally important class of semiconductor materials, primarily due to their attractive properties. They display superior ion transport, rapid electrode kinetics and high electrochemical behaviour towards water splitting and supercapacitance.^8,9^ Additionally, owing to their complex and elegant chemical composition, low band gap, valence states and stability, spinel ferrites have demonstrated interesting electrical, magnetic, optical, and catalytic properties.^10–15^ Spinel ferrites, such as NiFe_2_O_4_ and CoFe_2_O_4_, are composed of inexpensive, environmentally benign and easily accessible materials.^16–18^ Studies have indicated their applicability as electrode material in supercapacitors^19,20^ and lithium-ion batteries,^21,22^ as well as catalysts for electrochemical water splitting.^23,24^ Their electrochemical performance is ascribed to the presence of electrochemically active multivalent cations of Ni^3+^/Ni^2+^, Co^3+^/Co^2+^ and Fe^3+^/Fe^2+^. Despite all the appealing properties, the practical applications of these spinels for supercapacitors and water splitting are still relatively limited. This is due to unsatisfactory capacitance and a significant amount of energy would be inevitably lost due to internal resistances.^6,17^ Likewise, their use as bifunctional catalysts for overall water splitting is hampered by their limited activity,^25^ and the electrochemical performance of these materials still necessitates further improvement.

A promising approach to improving these spinel ferrite's electrochemical performance is to design a nanostructured solid solution. Consequently, the resultant solid solutions possess the additional composition-dependent synergistic effect of different elements, apart from properties emanating from the quantum confinement effects.^26^ In preparation of the solid solution, the chemical valence, crystallographic parameters, and radius of the component ions should be greatly considered for minimum formation energy and composition tuning in continuum.^27^ Interestingly, both NiFe_2_O_4_ and CoFe_2_O_4_ are isostructural and consist of isovalent Ni^2+^ and Co^2+^, thus they are characterized by the ability to form a highly diverse range of substitutional solid solutions following Hume-Rothery rules.^28^ The resultant effect is an enhancement of the overall properties as the mixed nickel–cobalt ferrite solid solution is an electrochemically efficient material compared to the pristine NiFe_2_O_4_ and CoFe_2_O_4_.

Likewise, controlled preparation of solid solutions is likely to augment the number of exposed atoms, surface area, number of active sites and electrical conductivity,^1^ which permit facile ion-diffusion and increased electrode–electrolyte interfacial interaction.^18,29,30^ This approach affords the fabrication of more sophisticated electrode materials for supercapacitors, HER and OER with enhanced activity.^31^ For example, nanospinel NiFe_2−*x*_Cr_*x*_O_4_ (0 ≤ *x* ≤ 1), prepared by a simple precipitation approach, showed an increase in OER activity when Cr content was increased from 0.2 to 1.0 mol in the spinel lattice of NiFe_2_O_4_.^32^ In addition, the synthesis of CuFe_2−*x*_Cr_*x*_O_4_ (0 ≤ *x* ≤ 1) has been achieved by employing a precipitation route, and the improvement in electronic properties was found in favour of OER catalysis in basic media.^33^

Nevertheless, for solid solutions, the composition control and nucleation in the nano regime are quite challenging. Particularly, the growth dynamics, solubility and crystallization process vary vastly when the component elements increase. In this regard, a well-designed reaction protocol and accurate control of the growth parameters are indispensable for obtaining homogeneous solid solution nanostructures and avoiding any probable phase segregation.^34^ Recently, solventless thermolysis has emerged as an alternative method for the large-scale fabrication of diverse homogeneous nanostructures. It is an environmentally benign, scalable and straightforward approach in which precursor materials undergo solid-state decomposition by thermal treatment.^35,36^ Compared with the most frequently used solution-based techniques, this approach ensures an inexpensive synthesis of nanoscopic oxide materials with surfaces free of any insulating surfactants. The surfactants adhere to the active surfaces of the catalyst and block/reduce the interaction of reactant molecules with the active sites. Therefore, the synthesis of bare surface nanoparticles may show much enhanced catalytic activity due to exposed active sites. Moreover, it has not been extensively utilized to synthesize nanoferrite solid solutions from metal–organic precursors.

Herein we have utilized the solventless thermolysis method to prepare uncapped solid solutions of Ni_1−*x*_Co_*x*_Fe_2_O_4_ (0 ≤ *x* ≤ 1) from the respective metal acetylacetonate precursors and examined their potential for electrochemical water splitting and supercapacitance. With reference to the review of existing published works, there is no report on the solventless synthesis of Ni_1−*x*_Co_*x*_Fe_2_O_4_ (0 ≤ *x* ≤ 1) solid solutions from metal acetylacetonates.

## 2. Experimental section

### 2.1 Chemicals

Nickel(ii) acetylacetonate (98%, Merck Schuchardt), cobalt(iii) acetylacetonate (98%, Merck-Schuchardt), iron(iii) acetylacetonate (97%, Sigma-Aldrich). All metal acetylacetonates complexes were used as received.

### 2.2 Solventless synthesis of Ni_1−*x*_Co_*x*_Fe_2_O_4_ (0 ≤ *x* ≤ 1) solid solutions

The Ni_1−*x*_Co_*x*_Fe_2_O_4_ (0 ≤ *x* ≤ 1) solid solutions of different stoichiometric compositions were prepared by solventless thermolysis of metal acetylacetonates. For the typical synthesis of ternary NiFe_2_O_4_ nanoparticles, 0.10 g (0.39 mmol) of nickel acetylacetonate and 0.27 g (0.78 mmol) of iron acetylacetonate were mixed and the solid mixture was grounded using pestle and mortar for ≈20 minutes to obtain a homogeneous mixture. The precursor mixture was then placed into a ceramic boat, which was placed in a reactor tube. The reactor tube was then introduced inside the carbolite tube furnace in such a way that the ceramic boat must be placed almost in the middle of the heating zone, followed by thermal treatment at 450 °C, at a heating rate of 20 °C min^−1^ for 1 h. After 1 h of annealing, the heating was switched off, and the furnace was left to cool naturally to ambient temperature. The reactor tube was taken out of the furnace upon cooling, and the product was collected for analysis without any post-treatment. Likewise, the synthesis of CoFe_2_O_4_ nanoparticles was achieved by employing similar procedures except that cobalt acetylacetonate was used instead of nickel acetylacetonate and the amount of cobalt and iron complexes were maintained in the same mole ratio of 1 : 2.

For the synthesis of quaternary Ni_1−*x*_Co_*x*_Fe_2_O_4_ (*x* = 0.2, 0.4, 0.6, 0.8) solid solutions, a known quantity of nickel acetylacetonate was partially substituted by appropriate amounts of cobalt acetylacetonate by adjusting the mole ratios of Co and Ni in the intervals of 0.2, 0.4, 0.6, and 0.8, while keeping the amount of iron acetylacetonate unchanged in the reaction mixture. The amounts of precursors used in each procedure are summarized in Table S1.† The reaction procedures for the entire series of solid solutions were kept similar to those employed to synthesize the ternary nickel and cobalt ferrites.

### 2.3 Instrumentation

The phase of the synthesized spinel Ni_1−*x*_Co_*x*_Fe_2_O_4_ (0 ≤ *x* ≤ 1) solid solutions was ascertained by powder X-ray diffraction (p-XRD) analysis employing a Bruker AXS D8 Advance X-ray diffractometer. The analysis was performed in the values of 2*θ* ranging from 10 to 80° and the data obtained were utilized to compute the lattice constants and crystallite size. Morphological characterization and analytical spectra of the nanoparticles was performed by a Zeiss Ultra Plus FEG Scanning Electron Microscope (SEM) equipped with an Oxford detector EDX at 20 kV using Aztec software for elemental analysis. The TEM and HRTEM imaging techniques were collectively used to determine the morphological features of the as-prepared Ni_1−*x*_Co_*x*_Fe_2_O_4_ (0 ≤ *x* ≤ 1) solid solutions. Imaging was performed on a JEOL 1400 TEM and JEOL 2100 HRTEM at accelerating voltages of 120 kV and 200 kV, respectively. The optical absorbance measurements were conducted in the UV-Vis spectral range on a Varian Cary 50 UV/Vis spectrophotometer. XPS analysis was conducted using Kratos Axis Ultra DLD spectrophotometer. For the experimental setup the emission was at 10 mA, the anode (HT) was 15 kV, the pressure for the analysis chamber was 5 × 10^−9^ torr, hybrid lens, and resolution to acquire survey scans was at 80 eV pass energy in slot mode centred at 597.5 with the width of 1205 eV, and steps at 1 eV and dwell time at 100 ms. High resolution core-level spectra were acquired at 40 eV pass energy in slot mode centred at 285 eV for C 1s, and the step size was 0.05 eV and dwell time at 500 ms.

### 2.4 Electrochemical studies

The electrochemical behaviour of Ni_1−*x*_Co_*x*_Fe_2_O_4_ (0 ≤ *x* ≤ 1) solid solutions was investigated by Gamry Potentiostat using a three-electrode system. A paste comprising Ni_1−*x*_Co_*x*_Fe_2_O_4_ sample (80 wt%), polyvinylidene difluoride (PVDF, 10 wt%) and acetylene black (10 wt%) was prepared using *N*-methyl pyrrolidinone (NMP) as a solvent. This paste was then applied to pre-cleaned and weighted nickel foam. The paste was then dried under a vacuum at 60 °C for 10 hours and used as a working electrode. A platinum wire and Hg/HgO were used as counter and reference electrodes, respectively. In all supercapacitance and water splitting experiments, 3 M and 1 M KOH were employed as the electrolyte, respectively. Measurements of charge storage capacity were performed by cyclic voltammetry (CV) at various scan rates as well as galvanostatic charge–discharge (GCD) at different current densities. Studies on the electrocatalytic activity of Ni_1−*x*_Co_*x*_Fe_2_O_4_ (0 ≤ *x* ≤ 1) electrodes were carried out by linear sweep voltammetry (LSV), chronoamperometry (CA) and cyclic voltammetry. For both OER and HER measurements, LSV was carried out at a scan rate of 2 mV s^−1^. Electrochemical impedance spectroscopy (EIS) was done in the frequency range of 0.05 Hz to 10 kHz with an applied AC amplitude of 10 mV.

## 3. Results and discussion

### 3.1 Powder X-ray diffraction analysis

Among different precursors for the preparation of metal oxides, the choice of metal acetylacetonate precursors is based on their low melting points, environmental benignity, commercial availability at affordable cost and their clean decomposition.^37^ Although they have been used to prepare spinel ferrites, Ni_1−*x*_Co_*x*_Fe_2_O_4_ (0 ≤ *x* ≤ 1) solid solution has not been prepared from metal acetylacetonates.

**Fig. 1:**
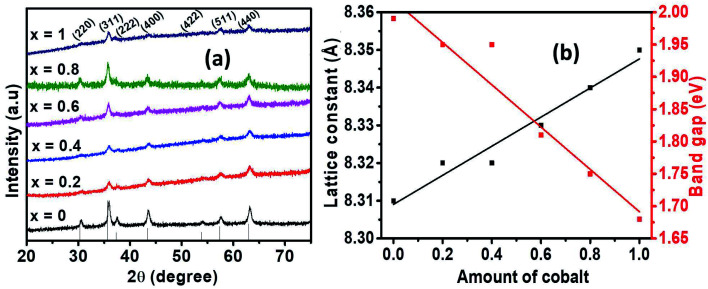
(a) p-XRD spectra of Ni_1−*x*_Co_*x*_Fe_2_O_4_ (0 ≤ *x* ≤ 1) series, (b) variation of lattice constant (left *y*-axis) and the optical band gap (right *y*-axis) of Ni_1*x*_Co_*x*_Fe_2_O_4_ (0 ≤ *x* ≤ 1) solid solutions.

The crystalline phase of Ni_1−*x*_Co_*x*_Fe_2_O_4_ (0 ≤ *x* ≤ 1) solid solutions was investigated using powder X-ray diffraction (p-XRD). Fig. 1(a) indicates the p-XRD spectra of Ni_1−*x*_Co_*x*_Fe_2_O_4_ solid solutions whose diffraction peaks are indexed to the (220), (311), (222), (400), (422), (511) and (440) planes, confirming the formation of a single-phase cubic spinels with *Fd*3*m* space group. The diffraction peaks of the pristine NiFe_2_O_4_ (*x* = 0) and CoFe_2_O_4_ (*x* = 1) were found to match well with the standard diffraction peaks of pure NiFe_2_O_4_ (ICDD # 00-044-1485) and CoFe_2_O_4_ (ICDD # 00-001-1121), respectively. Also, the diffraction peaks of the Ni_1−*x*_Co_*x*_Fe_2_O_4_ from *x* = 0.2 to *x* = 0.8 were consistent with those of the pristine materials and were found to lie in between the two pure ferrite systems of nickel and cobalt. Interestingly, no extra peaks associated with any impurity were present, suggesting the formation of a series of crystalline, monophasic solid solutions between pure NiFe_2_O_4_ and CoFe_2_O_4_. Similar diffraction patterns were obtained for pristine and alloyed compositions, which indicates that the lattice symmetry was retained during the substitution of the cations. It also indicates the successful inclusion of Co^2+^ into the NiFe_2_O_4_ lattice system. Additionally, the observed differences in peak intensities are ascribed to the internal stresses and planar faults caused by different amounts of Co^2+^ or uneven cation distribution at the tetrahedral and octahedral positions, respectively.^38^ Furthermore, slight peak broadening is observed in alloyed compositions, which could probably be caused by a reduction in crystallite size.^38,39^ Also, the broadening of the peak may be due to the increase in lattice strain arising from the presence of lattice defects or micro stresses, which is expected to increase with cobalt content in the pristine NiFe_2_O_4_.^38^

The lattice constants of the synthesized nanospinels were calculated from the p-XRD data and their values presented in Table 1. The estimation of the average values was achieved by considering the mean values of *a* computed for each diffraction peak by employing the equation: *a* = *dh*^2^ + *k*^2^ + *l*^2^, where *d* is the inter-planer spacing obtained from Bragg's law while h, k and l represent Miller indices.^40^ The lattice parameters are observed to increase almost in a linear fashion with the increasing amount of cobalt. The linear increase in lattice parameters can be explained by the difference in the ionic sizes of the substituting cations. The small Ni^2+^ ions (0.69 Å) are replaced by slightly larger Co^2+^ ions (0.74 Å) in the Ni_1−*x*_Co_*x*_Fe_2_O_4_ system, introducing strain and gradual expansion of the ferrite unit cell, which in turn contributes to the observed increase in the lattice parameters while the lattice symmetry remains intact. The values of lattice parameters for the alloyed samples were found to lie within the range of the lattice parameters of the two pristine cubic phases.^41^ The experimental values for lattice constant (8.313 Å) for pure NiFe_2_O_4_ are consistent with the standard values (8.337 Å, ICDD #: 00-044-1485). However, a slight discrepancy between the standard and experimental lattice constant values is noted due to the stresses and/or approximation that regard all ions to be rigid spheres spread in a rigid fashion.^42^ A plot of the lattice constants with respect to Co^2+^ content used in precursor mixture shows a linear relationship (Fig. 1(b)). In general, the observed linear dependence existing between the values of the lattice constant is in compliance with Vegard's law.^43^

**Table 1:** Lattice parameter (*a*), unit cell volume (*V*), band gap (*E*_g_) and EDX compositional analysis of Ni_1−*x*_Co_*x*_Fe_2_O_4_ solid solutions with variation in cobalt amounts (*x*)

Cobalt content (*x*)	Target ferrite composition	Stoichiometry obtained from EDX	Lattice constant (Å)	Crystallite size (nm)	Unit cell volume (Å^3^)	Band gap, *E*_g_ (eV)
0	NiFe_2_0_4_	Ni_1_._01_Fe_1.73_0_4.26_	8.313	19.40	574.48	1.99
0.2	Ni_0.8_Co_0.2_Fe_2_O_4_	Ni_0.74_Co_0.21_Fe_1.80_O_4.25_	8.317	8.91	575.31	1.95
0.4	Ni_0.6_Co_0.4_Fe_2_O_4_	Ni_0.58_Co_0.39_Fe_1.96_O_4.06_	8.321	14.43	576.14	1.95
0.6	Ni_0.4_Co_0.6_Fe_2_O_4_	Ni_0.39_Co_0.64_Fe_1.99_O_3.96_	8.334	10.73	578.84	1.81
0.8	Ni_0.2_Co_0.8_Fe_2_O_4_	Ni_0.29_Co_0.79_Fe_2.0_O_3.91_	8.349	12.90	581.97	1.75
1	CoFe_2_O_4_	Co_1.05_Fe_2.07_O_3.88_	8.351	9.68	582.39	1.67

The crystallite sizes of Ni_1−*x*_Co_*x*_Fe_2_O_4_ solid solutions were estimated from p-XRD data using Debye–Scherrer's formula.^44^ The estimation was done by considering the line broadening of the most intense peak (311), and the crystallite sizes of Ni_1−*x*_Co_*x*_Fe_2_O_4_ were found to be in the range of 8–19 nm (Table 1). In addition, the crystallite sizes were observed to decrease with an increasing amount of cobalt from *x* = 0 to *x* = 1 due to slight peak broadening and micro-strain in the lattice structure. However, a non-uniform trend in the changes of crystallite sizes with respect to Co^2+^ content was noted.

### 3.2 Analysis of chemical composition

**Fig. 2:**
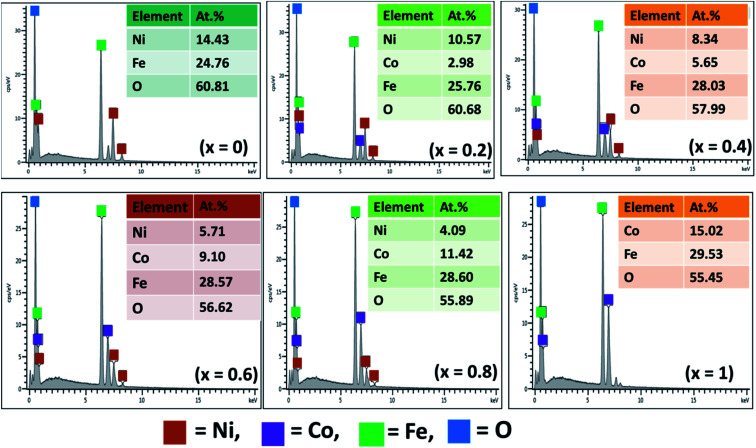
EDX spectra of Ni_1−*x*_Co_*x*_Fe_2_O_4_ (0 ≤ *x* ≤ 1) nanoparticles.

EDX analysis was performed to provide both qualitative and quantitative description and estimation of elemental composition in the synthesized Ni_1−*x*_Co_*x*_Fe_2_O_4_ solid solutions (Fig. 2). The EDX spectra of pristine NiFe_2_O_4_ (*x* = 0) authenticated the presence of Ni, Fe and O while that of CoFe_2_O_4_ (*x* = 1) confirmed the presence of Co, Fe and O. The EDX patterns of Ni_1−*x*_Co_*x*_Fe_2_O_4_ solid solutions from *x* = 0.2 to *x* = 0.8 confirmed the presence of Ni, Co, Fe and O. The experimental elemental composition in terms of normalized atomic percentage confirmed the presence of all atoms in a good stoichiometric ratio as expected. This infers that the nominal stoichiometric ratio of different metal components mixed at the time of preparation is consistent with the amount obtained in the final product (Table 1), suggesting no unexpected chemical reaction or any significant loss of ingredients. EDX elemental mapping of all the prepared spinel systems of Ni_1−*x*_Co_*x*_Fe_2_O_4_ signifies uniform distribution of the respective elements in the systems (Fig. S1†).

### 3.3 XPS analysis

**Fig. 3:**
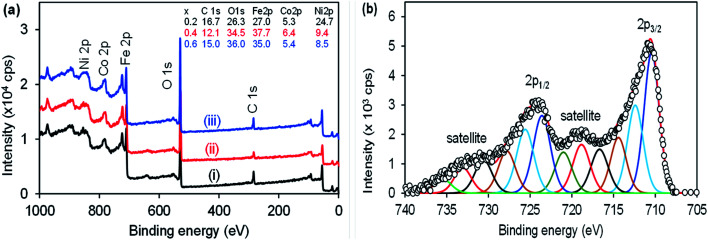
(a) Survey spectra for Ni_1−*x*_Co_*x*_Fe_2_O_4_, where *x* = (i) 0.2, (ii) 0.4 and (iii) 0.6. (b) High resolution core-level spectra of Fe 2p for Ni_1−*x*_Co_*x*_Fe_2_O_4_, where *x* = 0.6 and similar to the other samples.

The XPS was used to investigate the sample purity and to determine quantitative composition. The survey spectra in Fig. 3(a) shows that the samples Ni_1−*x*_Co_*x*_Fe_2_O_4_ contained the respective elements as per the prepared sample for *x* = (i) 0.2, (ii) 0.4 and (iii) 0.6. The samples contained Ni 2p (854 eV), Co (783 eV), Fe 2p (710 eV), and O 1s (529 eV). The elemental composition was as expected from the sample. However, the presence of C 1s (284 eV) was also noted, which is due to the acetylacetonate precursors used for the preparation of the ternary Ni_1−*x*_Co_*x*_Fe_2_O_4_ (*x* = 0.2, 0.4, 0.6, 0.8). The elemental composition of each sample is shown in the inset. The composition of Ni to Co was indicated by a subscript *x* during the preparation and from the XPS the sample with *x* = 0.2, Ni stoichiometric ratio was equivalent to the predicted, that is 0.86 (Ni) *vs.* 0.2 (Co). For *x* = 0.4, the Ni was 0.61 to 0.4 for Co and for *x* = 0.6, Ni was 0.42 and Co was 0.6. The results confirmed the increase in the amount of Co with the decreasing Ni content. The high resolution core-level spectra of Fe 2p in Fig. 3(b) is similar to the samples prepared. The fitting shows multiple oxidation states of Fe^0^, Fe^2+^ and Fe^3+^. The high resolution of core-level spectra of Co 2p and Ni 2p could not be deconvoluted due to very small signals. The high resolution core-level spectra of O 1s and C 1s shown in the electronic support information, Fig. S2† of the ternary Ni_1−*x*_Co_*x*_Fe_2_O_4_ (*x* = 0.4) was similar for other samples (*x* = 0.2 and 0.4). The spectra were deconvoluted and multiple peaks were identified, corresponding to the material used for the synthesis of Ni_1−*x*_Co_*x*_Fe_2_O_4_ at 450 °C.

### 3.4 SEM, TEM and HRTEM analysis

**Fig. 4:**
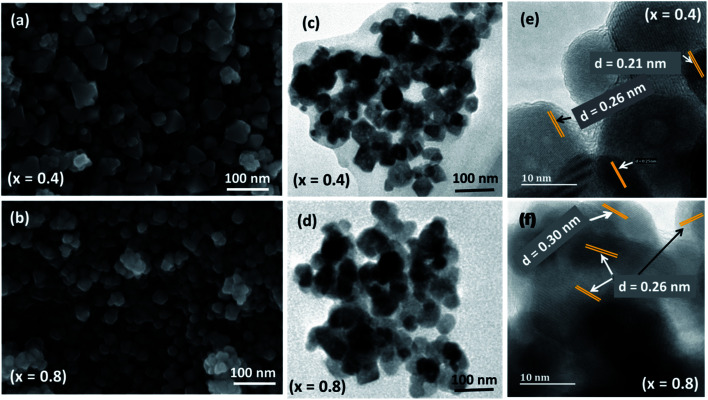
Representative (a and b) SEM, (c and d) TEM, and (e and f) HRTEM images of Ni_1−*x*_Co_*x*_Fe_2_O_4_ (*x* = 0.4 and 0.8) solid solutions.

The SEM micrograph presented in Fig. 4(a and b) and S3† show the surface morphology of Ni_1−*x*_Co_*x*_Fe_2_O_4_ solid solutions prepared from molecular precursors of metal acetylacetonates by using the melt method. It is apparent from the SEM images that the formation of the octahedron and cubic-shaped particles, with some truncated edges, of monophasic Ni_1−*x*_Co_*x*_Fe_2_O_4_ solid solutions was achieved at different Co^2+^ stoichiometries. It can also be inferred from the SEM images that the uniformity of particles varied with different quantities of cobalt incorporated in the crystal lattice of nickel ferrite. This variation in particle uniformity is due to the difference between the driving force responsible for particle boundary movement and the retarding force exerted by the pores during particle growth/formation.^45^ The appearance of somewhat agglomerated Ni_1−*x*_Co_*x*_Fe_2_O_4_ nanoparticles is a result of interaction arising from the magnetic nature of the nanoparticles, which make them to be held together due to intensive van der Waals attractive force.^46^ Particle agglomeration can also be ascribed to the absence of capping agents during thermolysis, as the method proceeds without the use of solvents or passivating agents. The average particle size estimated from SEM micrographs (47.8 nm) was larger than the value of crystallite size computed according to the Scherrer formula from p-XRD data (12.7 nm). However, SEM analysis has its limitations in image magnifications and in addition, this disparity in particle size between p-XRD and SEM could probably be attributable to the aggregation of the nanoparticles.^46^

In order to have a better understanding of the size and morphology, the Ni_1−*x*_Co_*x*_Fe_2_O_4_ (0 ≤ *x* ≤ 1) solid solutions were also analyzed by TEM analysis (Fig. 4(c and d) and S4†). Well-separated cubic and octahedron-shaped nanoparticles are observed, having an average size around 14–23.2 nm. We can also observe that some particles are stuck together by interfacial forces, which are considered to be the cause for somewhat agglomerated particles. Such aggregation of particles has also been reported in the literature.^47^ The average particle size of Ni_1−*x*_Co_*x*_Fe_2_O_4_ (0 ≤ *x* ≤ 1) solid solutions observed in the TEM images is consistent with those estimated from p-XRD. The representative HRTEM image presented in Fig. 4(e and f) and those shown in Fig. S5† clearly display the interplanar lattice spacing, which shows that extremely fine particles are properly crystallized into single crystals. The average *d*-spacing of 0.21, 0.25, 0.29 and 0.30 nm were computed by profile of frame at different regions of the image. The interplanar distances are in compliance with (400), (311) and (220) planes of the spinel NiFe_2_O_4_ and CoFe_2_O_4_ nanospinels.^48,49^ The absence of a secondary phase in HRTEM analysis suggests a good agreement with the p-XRD results.

### 3.5 Analysis of optical properties

**Fig. 5:**
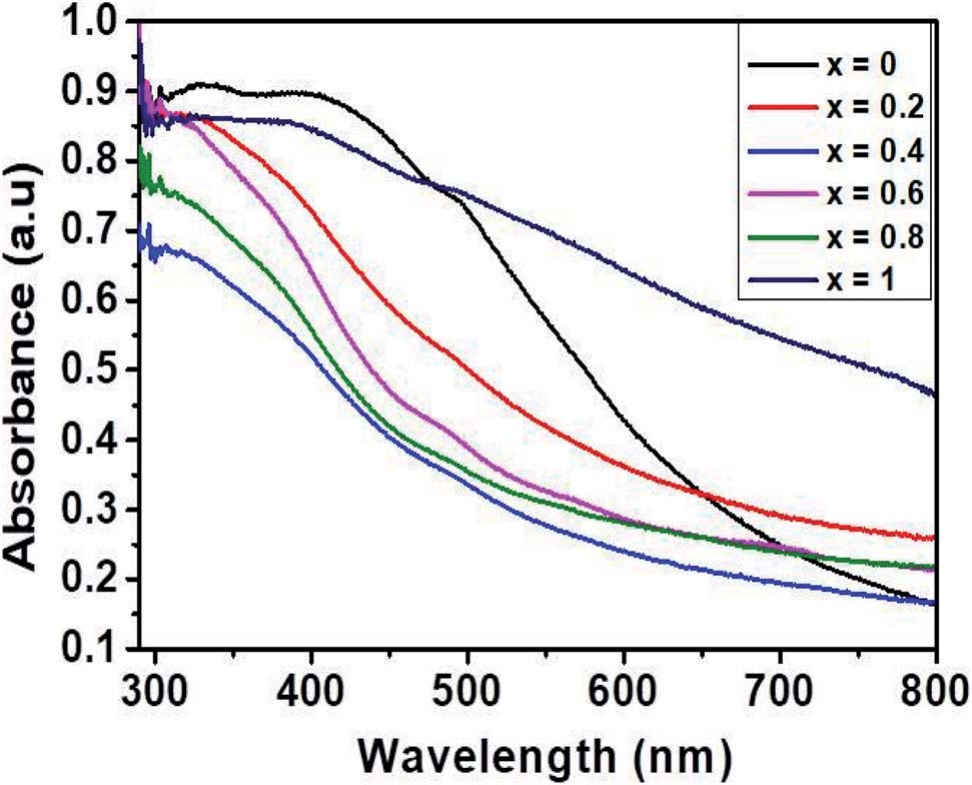
UV-Vis absorption spectrum of Ni_1−*x*_Co_*x*_Fe_2_O_4_ (0 ≤ *x* ≤ 1) solid solutions.

The optical properties of the synthesized Ni_1−*x*_CoxFe_2_O_4_ solid solutions were studied by UV-Vis spectroscopy, and the results obtained are displayed in Fig. 5. The absorption spectra of the prepared solid solution series show an absorption in the range of 400–800 nm. The values of band gap were computed from the Tauc plots of (*αhν*)^2^*vs.* photon energy, *hν* (Fig. S6†), where *α* stands for absorption coefficient, *ν* represents the frequency of UV-Vis radiation, and *h* is Planck's constant.^50^ The estimated values of band gap (Table 1) were found to decrease slightly from 1.98 to 1.67 eV with the increase in cobalt content (Fig. 1(b)), and is related to the inclusion of slightly larger Co^2+^ ions in NiFe_2_O_4_ which creates less deep Co^2+^ states due to weaker electrostatic interaction, hence shortening the energy band gap.^51^ To a certain degree, the change in the energy band gap might also be affected by localized electronic states present in the material. In the present case, the redshift is attributed to the sp–d exchange interactions occurring between the band electrons and the localized d electrons of Co^2+^ replacing Ni^2+^.^52^ A similar observation in the absorption edge was previously reported in copper substituted nickel ferrite.^53^ In general, it is worth noting that the range of band gap values obtained in this study suggests the applicability of the synthesized Ni_1−*x*_Co_*x*_Fe_2_O_4_ solid solutions in photocatalysis and optoelectronics.

### 3.6 Supercapacitance

**Fig. 6:**
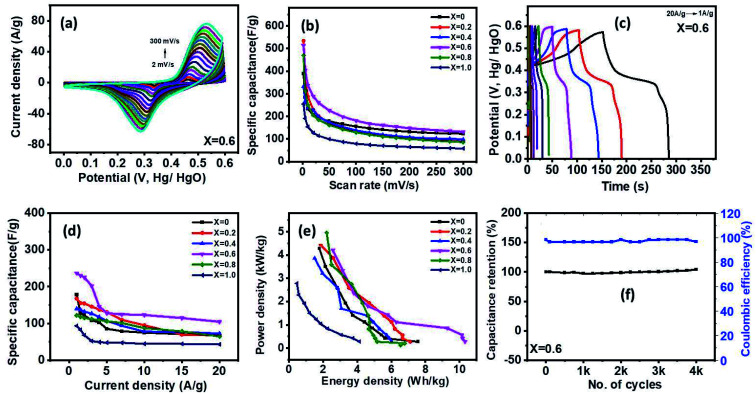
(a) CV graphs of the *x* = 0.6 electrode for various scan rates (2–300 mV s^−1^), (b) specific capacitance *versus* scan rate for various samples, (c) GCD graphs of the *x* = 0.6 electrode for various current densities (1–20 A g^−1^), (d) specific capacitance *versus* current density for various samples, (e) variation of energy and power density for various samples, and (f) stability tests for the *x* = 0.6 electrode.

The supercapacitor characteristics of the Ni_1−*x*_Co_*x*_Fe_2_O_4_ (0 ≤ *x* ≤ 1) electrodes were evaluated using cyclic voltammetry (CV) and galvanostatic charge–discharge (GCD) curves in 3 M KOH electrolyte within a potential range (0–0.6 V *vs.* Hg/HgO). Fig. 6(a) displays the CV graphs of Ni_1−*x*_Co_*x*_Fe_2_O_4_ samples with compositions *x* = 0.6 from a low scan rate (2 mV s^−1^) to a high scan rate (300 mV s^−1^). The CV graphs of other Ni_1−*x*_Co_*x*_Fe_2_O_4_ electrodes with *x* = 0, 0.2, 0.4, 0.8 and 1 are indicated in the ESI, Fig. S7.† In all compositions of Ni_1−*x*_Co_*x*_Fe_2_O_4_ samples, two peaks generated by reversible redox reaction were observed in the CV plots, indicating the pseudocapacitive property of the synthesized solid solutions. Also, a shift in redox peak towards higher potential with a change in the scan rates was observed, which suggests that a diffusion-controlled charge-transfer process is the predominant charge-transport process.^54^ As seen in the graph, the shape of the CV curves remained unchanged even at a high scan rate, signifying excellent capacitive, stability, and charge storing properties of the electrodes even at the fast charge-transfer process.^55^ The quantitative analysis of the electrochemical performance of Ni_1−*x*_Co_*x*_Fe_2_O_4_ (0 ≤ *x* ≤ 1) working electrodes was carried out by deducing the specific capacitance from the CV data using eqn (1) given below:1
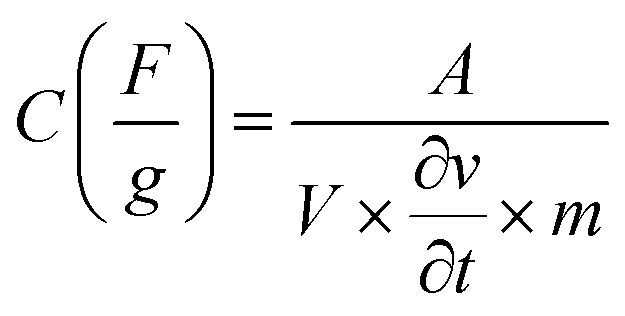
where *A* denotes the area under the CV curve, *V* stands for the potential window, 
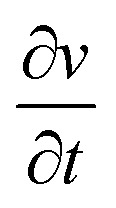
 is the scan rate, and *m* is the mass of the Ni_1−*x*_Co_*x*_Fe_2_O_4_ electrodes. Fig. 6(b) presents the voltammograms showing the variation of specific capacitance (F g^−1^) with scan rates (mV s^−1^). The trend of the results shows that the specific capacitance of all samples decreases as the scan rate is amplified from 2 to 300 mV s^−1^. The observed high specific capacitance at lower scan rates can be explained by easy diffusion and high mobility of the electrolyte ions into the active material. There is more time available to count for enhanced interaction between the electrolyte ions and the ions of deposited electrically active material at a lower scan rate. Increasing the scan rate to higher values leads to the reduction in the specific capacitance because there is not enough time for the ions in motion to fill the spaces of active material, resulting in limited interactions on the outer surfaces only. Consequently, at higher scan rates, some active parts of the surface areas become unavailable for charge storage.^56^ The values of specific capacitance obtained at 2 mV s^−1^ are 388, 534, 332, 513, 470 and 254 F g^−1^ for *x* = 0, 0.2, 0.4, 0.6, 0.8 and 1, respectively. It is obvious that at the lowest scan rate of 2 mV s^−1^, the compositions *x* = 0.2 and 0.6 recorded higher specific capacitance than other compositions, particularly the pristine NiFe_2_O_4_. This might be caused by the appreciable content and uniform distribution of Co^2+^ ions in the NiFe_2_O_4_ lattice. The distribution of Co^2+^ ions in the octahedral sites causes a change in the lattice parameters and bond length due to the ionic size difference between Co^2+^ and Ni^2+^ ions. Consequently, stronger interaction between Co^2+^ and O^2−^ ions occurs, leading to the splitting of degenerate orbitals, and when there are more chances for the working electrode to react with the electrolyte, the increase in specific capacitance becomes obvious.^57^

The galvanostatic charge–discharge studies of Ni_1−*x*_Co_*x*_Fe_2_O_4_ samples conducted at different charge–discharge current densities (1–20 A g^−1^) are presented in Fig. 6(c) and S8.† As seen in the charge–discharge curves at various current densities, an apparent plateau and nonlinearity were observed, suggesting pseudo-capacitance behaviour of the Ni_1−*x*_Co_*x*_Fe_2_O_4_ electrode materials with various stoichiometric compositions. Among all the compositions investigated, nanospinel ferrite electrodes with *x* = 0.6 demonstrated a longer charge–discharge time, signifying superiority in charge storage capacity. The variation of specific capacitance with respect to current density for Ni_1−*x*_Co_*x*_Fe_2_O_4_ solid solutions is displayed in Fig. 6(d). The obtained specific capacitance was derived from eqn (2) below.2
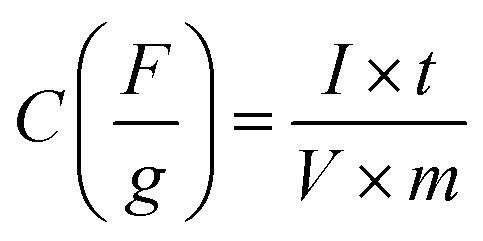
where *I* is the discharge current, *t* is the discharge time, *V* is the applied potential window, and *m* is the mass of Ni_1−*x*_Co_*x*_Fe_2_O_4_ samples. The specific capacitances of 178, 168, 140, 237, 121 and 93 F g^−1^ were obtained for electrodes with *x* = 0, 0.2, 0.4, 0.6, 0.8 and 1, respectively, at current density of 1 A g^−1^. The specific capacitance of the electrodes decreased to 70, 68, 73, 105, 65 and 43 F g^−1^ for the electrodes with *x* = 0, 0.2, 0.4, 0.6, 0.8 and 1, respectively, at current density of 20 A g^−1^. The electrodes retained 39.3, 40.5, 52.1, 44.3, 53.3, and 46.2% of their charge storage capacity on increase current density from 1 to 20 A g^−1^. Our results suggest that these electrodes have a good rate capability and could be used in fast-charging devices. The best performance was observed for *x* = 0.6, which exhibited a specific capacitance of 237 F g^−1^ at a current density of 1 A g^−1^. The overall trend shows a decrease in specific capacitance with the increase in current density and is ascribed to the limits in diffusion movements of electrolyte ions.^58^ The inner active sites of nanoscale Ni_1−*x*_Co_*x*_Fe_2_O_4_ electrodes at low current densities are fully utilized due to low ohmic drop, which offers enough time for redox reactions, resulting in the high specific capacity. However, the high charge–discharge rate at high current densities presents an inevitable time constraint making it difficult to maintain high capacities. Also, at this point, the movement of ions in the electrolyte is dependent on diffusion, and the charge storage center is limited on the outer surface.^59^ The superior supercapacitor performance of the Ni_1−*x*_Co_*x*_Fe_2_O_4_ samples was compared with other studies on binary and ternary metal oxides for supercapacitor applications. Bhujun group employed a sol–gel method to synthesize ternary transition metal ferrites of NiCoFe_2_O_4_, NiCuFe_2_O_4_, and CuCoFe_2_O_4_, which acquired the maximum specific capacitance of 50, 44, 76.9 F g^−1^, respectively at the current density of 1 A g^−1^.^16^ In addition, binary transition metal oxide, NiMnO_3_ synthesized *via* a hydrothermal route recorded a specific capacitance of 230 F g^−1^ at 1 A g^−1^.^60^ When compared with other binary/ternary metal oxides, the nanospinel Ni_1−*x*_Co_*x*_Fe_2_O_4_ electrode with composition *x* = 0.6 synthesized by solventless thermolysis method showed an excellent electrochemical performance. It is also believed that Co^2+^ tends to offer additional holes while Ni^3+^ provides extra electrons in the redox reactions, thus enhancing conductivity and capacitive performance. Further comparison of the specific capacitance of Ni_0.4_Co_0.6_Fe_2_O_4_ electrode obtained in this study with other previously reported metal oxide-based electrodes is provided in Table S2.†

The relationship between energy and power density for all the samples is shown in Fig. 6(e). The energy (*E*) and power (*P*) density was obtained from the GCD measurement by using eqn (3) and (4), respectively, where *C* is the capacitance of the electrode, *V* is the applied window potential (volt), and *t* is the time (seconds).3
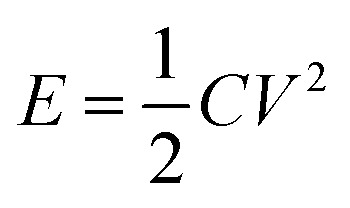
4
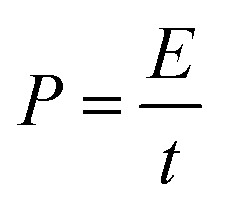


Among all the samples examined, the nanoscopic Ni_1−*x*_Co_*x*_Fe_2_O_4_ electrode with *x* = 0.6 displayed a superior energy density of 10.3 W h kg^−1^ while also showing a high-power density with a peak value of 4208 (W kg^−1^). The energy and power densities obtained in this study are superior to the Ni_1−*x*_Co_*x*_Fe_2_O_4_ nanoparticles prepared hydrothermally by Sharifi *et al.*^57^ The long-term stability test for Ni_1−*x*_Co_*x*_Fe_2_O_4_ electrode with *x* = 0.6 shown in Fig. 6(f) was conducted up to 4000 cycles at current density 7 A g^−1^. The results showed about 100% retention in the charge storage capacity at the end of 4000 cycles of charge–discharge study with about 97% coulombic efficiency. Other compositions of Ni_1−*x*_Co_*x*_Fe_2_O_4_ samples also showed high charge retention with high coulombic efficiency up to 4000 cycles of charge–discharge study (Fig. S9†). Based on CV, GCD, and stability tests, the *x* = 0.6 electrode shows the highest performance for the supercapacitor electrode. These results might be due to the careful consideration of surface area, porosity, and conductivity of the electrode.

### 3.7 Hydrogen evolution reaction (HER)

**Fig. 7:**
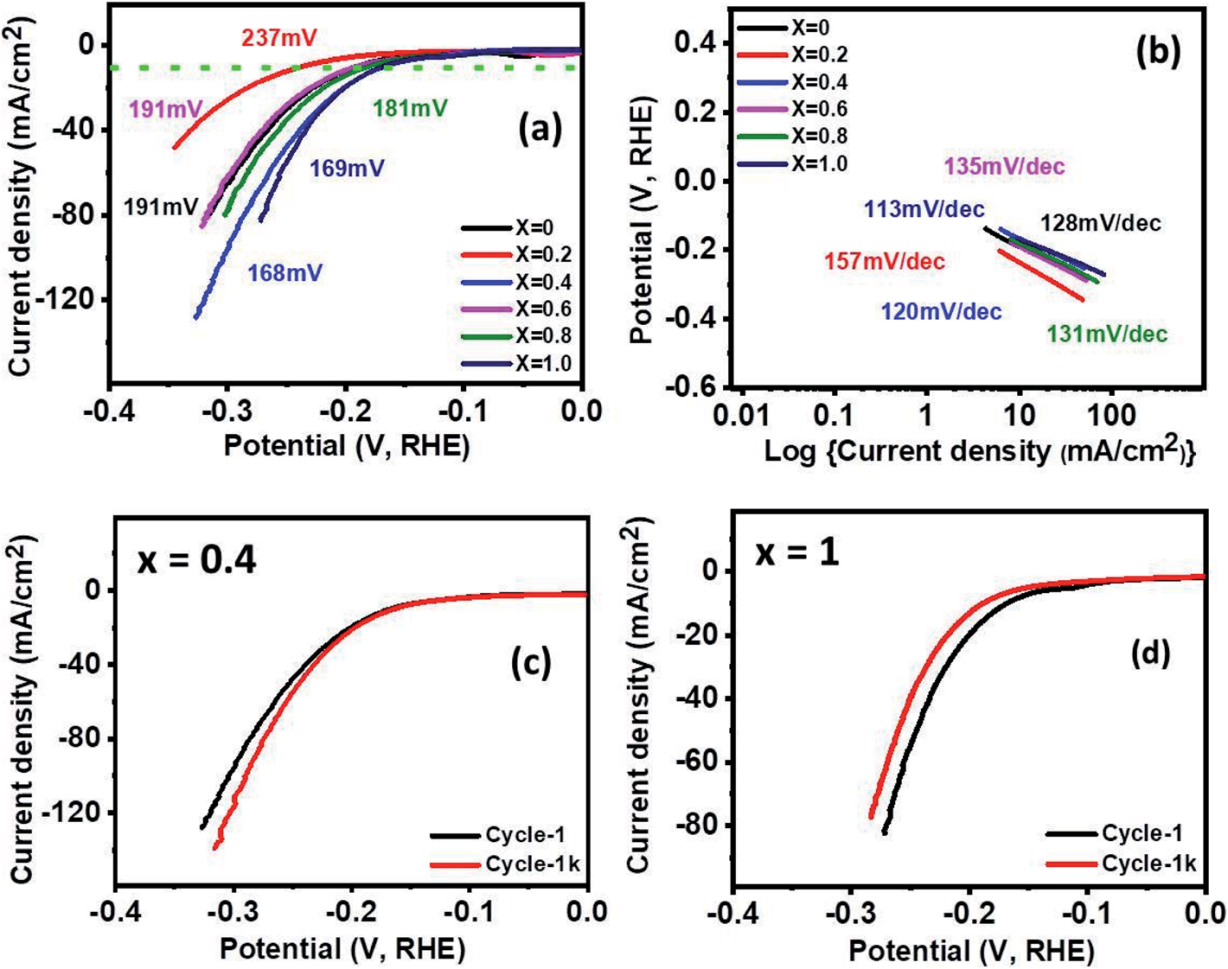
(a) HER polarization curves, (b) Tafel slopes for different samples, (c) HER polarization curves at various cycles for the *x* = 0.4, and (d) HER polarization curves at various cycles for *x* = 1.0.

The electrocatalytic behavior of the Ni_1−*x*_Co_*x*_Fe_2_O_4_ (0 ≤ *x* ≤ 1) electrodes for efficient HER was examined using linear sweep voltammetry (LSV) in 1 M KOH. As seen in Fig. 7(a), the low overpotentials of 191, 237, 168, 191, 181 and 169 mV were required for *x* = 0, 0.2, 0.4, 0.6, 0.8, and 1 electrodes, respectively to deliver the current density 10 mA cm^−2^. All results showed very low overpotentials, but among them, *x* = 0.4 (168 mV) and *x* = 1 (169 mV) showed fairly low overpotentials, indicating better catalytic activity than other samples. There are many reports regarding the synthesis of efficient HER catalysts by using Ni, Fe, and Co, which are relatively cheap and abundant on earth, instead of expensive noble metal materials. For example, Adamson's group synthesized the Co–Fe binary metal oxide electrocatalyst, which exhibited the overpotential value of 220 mV at a current density 10 mA cm^−2^, signifying much higher activity than CoO (387 mV) and Fe_3_O_4_ (431 mV) at 10 mA cm^−2^.^61^ Also, nanostructured flower-like nickel–cobalt oxide was synthesized by the Elakkiya group. It demanded the overpotential of 370 mV at 10 mA cm^−2^, which is higher activity than NiO and Co_3_O_4_ comprising the nickel–cobalt oxide nanomaterials.^62^ Compared with other group's work, the multicomponent Ni_1−*x*_Co_*x*_Fe_2_O_4_ (*x* = 0.4 and 1) electrodes having low overpotential and high current density show high electrocatalytic activity for hydrogen evolution. Table S3† shows a detailed comparison of HER performance of the synthesized Ni_1−*x*_Co_*x*_Fe_2_O_4_ (*x* = 0.4 and 1) with other reported Ni/Co-based electrocatalysts in alkaline electrolytes. Fig. 7(b) shows the Tafel slope, an indicator of electrocatalytic kinetics. It was plotted with the aid of the equation *η* = *a* + *b* log *j*; where *η* is the overpotential, *a* is a constant, *b* is the Tafel slope, and *j* is the current density. The calculated Tafel slopes are 128, 157, 120, 135, 131 and 113 mV dec^−1^ for electrodes with compositions *x* = 0, 0.2, 0.4, 0.6, 0.8, and 1, respectively. Since the lower Tafel slope indicates the faster kinetics, thus electrodes with molar ratios *x* = 0.4 (120 mV dec^−1^) and *x* = 1 (113 mV dec^−1^) exhibit better reaction kinetics than other samples. In Fig. 7(c and d), stability tests of the samples with *x* = 0.4 and *x* = 1 were performed by comparing 1^st^ polarization curve with the 1000^th^ polarization curve. Even after 1000 cycles of cyclic voltammetry, a little deviation was observed for both graphs, indicating high durability. Similarly, after 1000 cycles of CV measurements, other samples demonstrated high stability, which is consistent with or slightly different from the first graph (Fig. S10†). For efficient electrocatalysis, the electrode material needs to have low overpotential and Tafel slope with high stability. The Ni_1−*x*_CoxFe_2_O_4_ electrodes with *x* = 0.4 and *x* = 1, prepared by the solventless method, possess these crucial elements and show favourable electrocatalytic properties for HER. In addition, the effect of electronic push in cobalt substituent on the HER performance of Ni-based materials has been established, where partial electrons adjacent to nickel sites are pushed by cobalt substituent resulting in an increase in the number of lattice O^2−^ groups and consequently boosting H^+^ adsorption and charge transfer for the HER.^63^

### 3.8 Oxygen evolution reaction (OER)

**Fig. 8:**
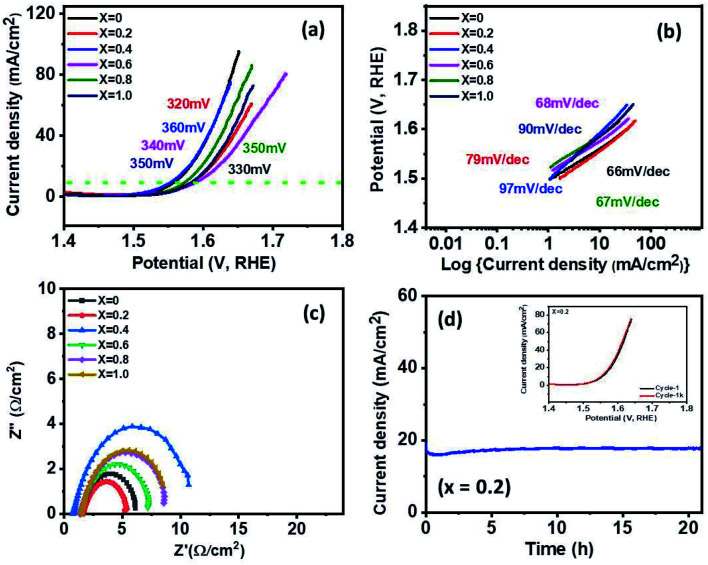
(a) OER polarization curves, (b) Tafel slopes for various samples, (c) electrochemical impedance spectroscopic spectra of all the samples various potentials (*vs.* SCE) at 0.5 V, and (d) chronoamperometry characteristics of the *x* = 0.2 in 1 M KOH (inset) OER polarization curves at various cycles for the *x* = 0.2.

The electrocatalytic activity of Ni_1−*x*_Co_*x*_Fe_2_O_4_ (0 ≤ *x* ≤ 1) electrodes for OER was analyzed using LSV, electrochemical impedance spectroscopy (EIS), and chronoamperometric (CA) measurements in 1 M KOH. At the current density of 10 mA cm^−2^, the overpotentials of 330, 320, 360, 340, 350 and 350 mV were observed along with low Tafel slopes of 66, 79, 97, 68, 67 and 90 mV dec^−1^ for *x* = 0, 0.2, 0.4, 0.6, 0.8 and 1, respectively as shown in Fig. 8(a and b). The values of the Tafel slope give an insight into the kinetics of the OER mechanism. The electrode with a lower Tafel slope is expected to display faster reaction kinetics that accelerates higher OER activity. The *x* = 0.2 electrode exhibited a lower overpotential of 320 mV with a low Tafel slope of 79 mV dec^−1^. The greater OER activity demonstrated by Ni_1−*x*_Co_*x*_Fe_2_O_4_ electrode with *x* = 0.2 can also be ascribed to the multicomponent structure and higher number of active sites because of the small crystallite size (8.91 nm) compared to other compositions. This surpasses the results of oxygen evolution activity obtained in other studies based on Co, Fe, Ni transition metal materials such as NiCo_2_O_4_ nanoneedles (565 mV @ 10 mA cm^−2^ overpotential, 292 mV dec^−1^ Tafel slope), NiCo_2_O_4_ nanosheets (888 mV @ 10 mA cm^−2^, 393 mV dec^−1^),^64^ N-doped graphene-NiCo_2_O_4_ hybrid paper (434 mV @ 10mA cm^−2^, 156 mV dec^−1^ Tafel slope),^65^ and Co_0.5_ Fe_0.5_ S@N-MC (410 mV @ 10 mA cm^−2^, 159 mV dec^−1^ Tafel slope).^66^ Table S4† shows a comparison of OER performance of the synthesized Ni_0.8_Co_0.2_Fe_2_O_4_ with other reported Ni/Co-based electrocatalysts in alkaline electrolytes. The Nyquist plots, shown in Fig. 8(c), were studied from the electrochemical impedance spectroscopy in the frequency range of 0.05 Hz to 10 kHz with an applied AC amplitude of 10 mV. The intersection value of the real axis represents electrolyte resistance. The low electrolyte resistance of ∼1.5 Ω cm^−2^ was observed in the graph. Furthermore, all graphs appear semi-circle at 0.5 V potential (V *vs.* Hg/HgO). The semi-circle shown in the graph indicates the charge transfer resistance at the interface between 1 M KOH electrolyte and the Ni_1−*x*_Co_*x*_Fe_2_O_4_ electrode. The lower the diameter of the semi-circle suggests the less charge transfer resistance. The observed values of the resistance are ∼4.6, 3.5, 10, 5.8, 7.2, 7 Ω cm^−2^ for *x* = 0, 0.2, 0.4, 0.6, 0.8 and 1, respectively. Among the stoichiometric molar ratios of the spinel Ni_1−*x*_Co_*x*_Fe_2_O_4_ electrodes, an electrode with composition *x* = 0.2 displayed low charge transfer resistance (3.5 Ω cm^−2^), representing better OER catalytic properties than other compositions. Long-term stability and durability tests were conducted by 1000 cycles of CV measurements and chronoamperometry for *x* = 0.2, as presented in Fig. 8(d). The *x* = 0.2 electrode delivered a stable high current density of 18 mA cm^−2^ over 20 h at a constant voltage of 0.55 V. In addition, the polarization curve of *x* = 0.2 shows a perfect match between the 1^st^ cycle and the 1000^th^ cycle (inset). In Fig. S11,† other samples also showed a nearly identical graph between the 1^st^ and 1000^th^ cycle and stable high current density over 20 h at a constant voltage of 0.55 V, but a little fluctuation was observed in some graphs, which indicate oxygen gas during oxygen evolution.

## 4. Conclusion

Summarily, the advantages of eco-friendliness, simplicity, and scalability of the solventless method have been exploited to synthesize homogeneous nanoscale Ni_1−*x*_Co_*x*_Fe_2_O_4_ (0 ≤ *x* ≤ 1) solid solutions. The p-XRD analysis confirmed the formation of a series of single-phase cubic spinel ferrites with space group *Fd*3*m*. It was observed that the nanospinel Ni_0.4_Co_0.6_Fe_2_O_4_ electrode demonstrated a longer charge–discharge time, signifying superior charge storage capacity. For efficient HER electrocatalysis, the Ni_0.6_Co_0.4_Fe_2_O_4_ and CoFe_2_O_4_ electrodes showed low overpotential and Tafel slope and high stability, which are crucial elements for HER. Similarly, Ni_0.8_Co_0.2_Fe_2_O_4_ exhibited a lower overpotential of 320 mV with a low Tafel slope of 79 mV dec^−1^, indicating enhanced OER activity. The results in this study affirmed that the synergism between nickel and cobalt in the crystal lattice of spinel nickel ferrite has a tremendous influence on the electrochemical performance of the resultant Ni_1−*x*_Co_*x*_Fe_2_O_4_ solid solution for energy conversion and storage.

## Conflicts of interest

The authors declare no conflict of interest.

## Supplementary Material

RA-011-D1RA04833C-s001
