# Supplementary material for: Solventless synthesis of nanospinel Ni1−xCoxFe2O4 (0 ≤ x ≤ 1) solid solutions for efficient electrochemical water splitting and supercapacitance
Source: RSC Adv. 2021 Sep 20;11(49):31002–14. doi: 10.1039/d1ra04833c (PMC9041409; doi:10.1039/d1ra04833c)
Supplement: RA-011-D1RA04833C-s001 [file RA-011-D1RA04833C-s001.pdf]

Electronic Supplementary Material (ESI) for RSC Advances.
This journal is © The Royal Society of Chemistry 2021

Supporting Information

Solventless synthesis of nanospinel $Ni_{1-x}Co_xFe_2O_4$ ($0 \leq x \leq 1$) solid solutions for efficient electrochemical water splitting and supercapacitance

Nyemaga Masanje Malima,[1,2] Malik Dilshad Khan,1,3* Jonghyun Choi,4 Ram K. Gupta4 Philani Mashazi,[5,6] Tebello Nyokong[6] and Neerish Revaprasadu[1]*

[1]Department of Chemistry, University of Zululand, Private Bag X1001, KwaDlangezwa 3880, South Africa.

[2]Department of Chemistry, College of Natural and Mathematical Sciences, University of Dodoma, P.O. Box 338, Dodoma, Tanzania.

[3]Institute of Physical Chemistry, Polish Academy of Sciences, Kasprzaka 44/52, 01-224 Warsaw, Poland.

[4]Department of Chemistry, Pittsburg State University, Pittsburg, KS, 66762, USA.

[5]Department of Chemistry, Rhodes University, P.O Box 94, Makhanda, 6140, South Africa.

[6]Institute for Nanotechnology Innovation, Rhodes University, P.O Box 94, Makhanda, 6140, South Africa.

***Email**: RevaprasaduN@unizulu.ac.za; malikdilshad@hotmail.com

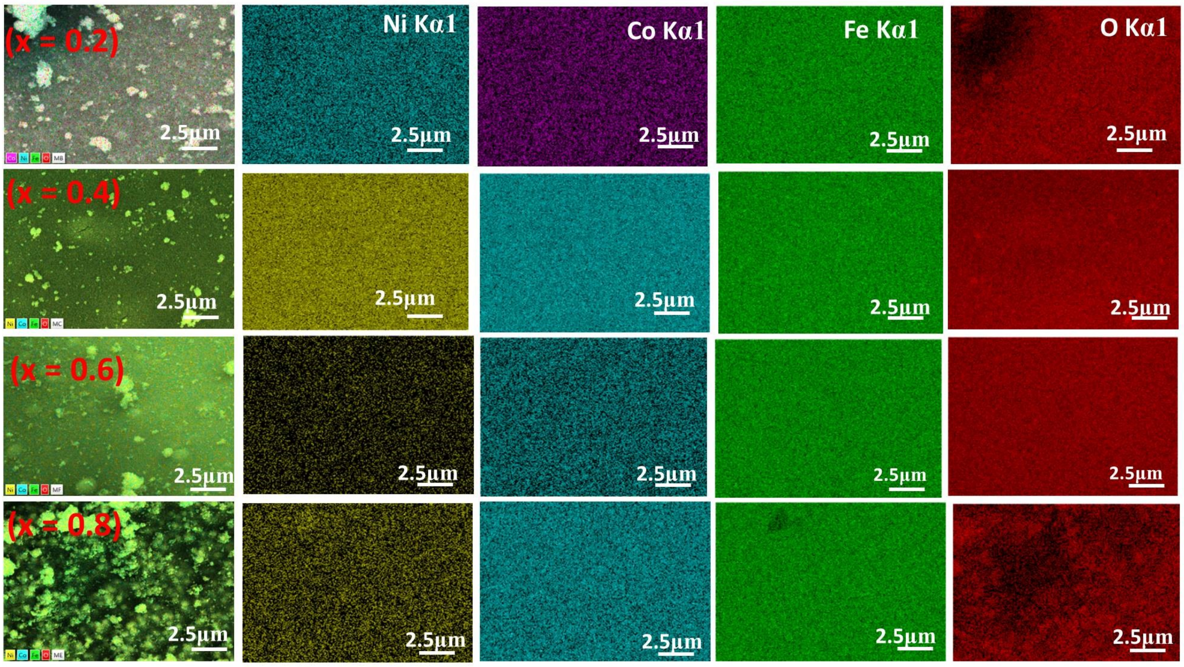

Fig. S1. EDX elemental mapping of $Ni_{1-x}Co_xFe_2O_4$ ($0.2 \leq x \leq 0.8$) solid solutions showing a uniform distribution of elements.

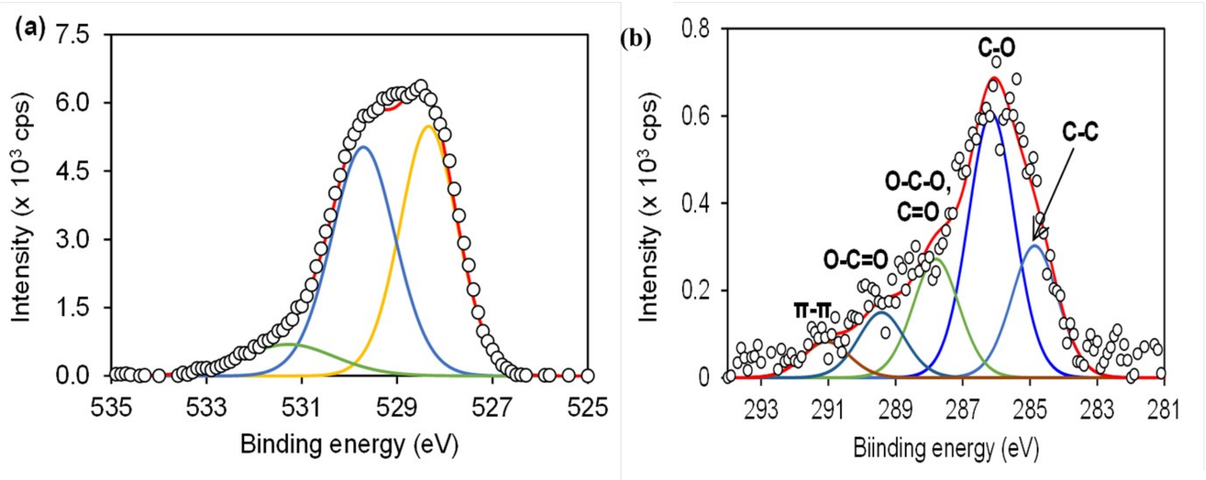

Fig. S2. XPS High resolution core-level spectra of (a) O 1s and (b) C 1s of the ternary $Ni_{1-x}Co_xFe_2O_4$ (x = 0.4).

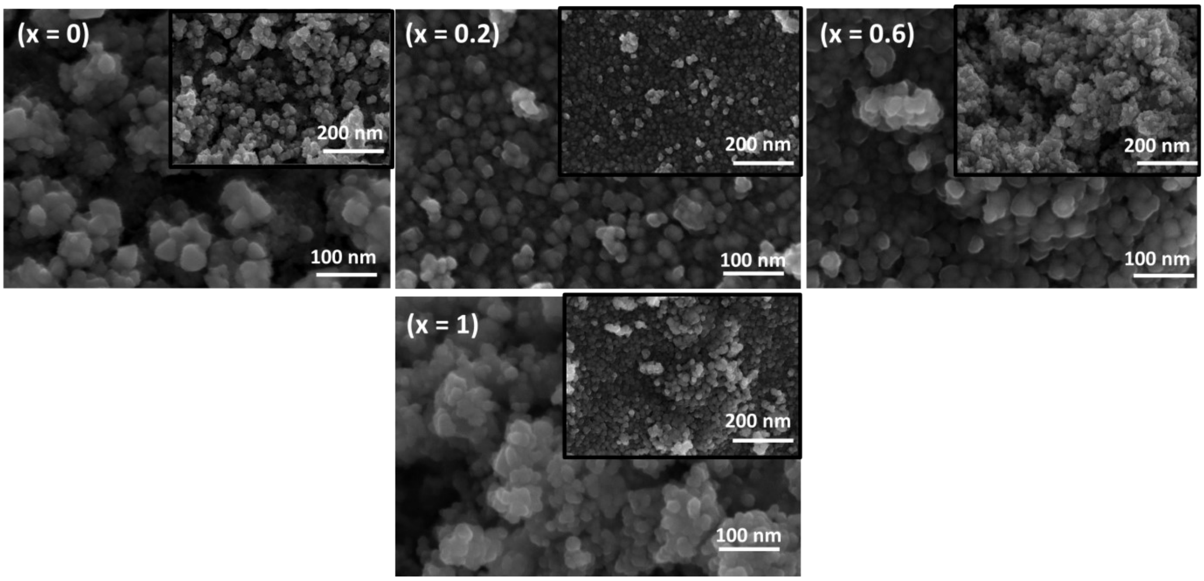

Fig. S3. SEM images of $Ni_{1-x}Co_xFe_2O_4$ (0, 0.2, 0.6 and 1) nanoparticles.

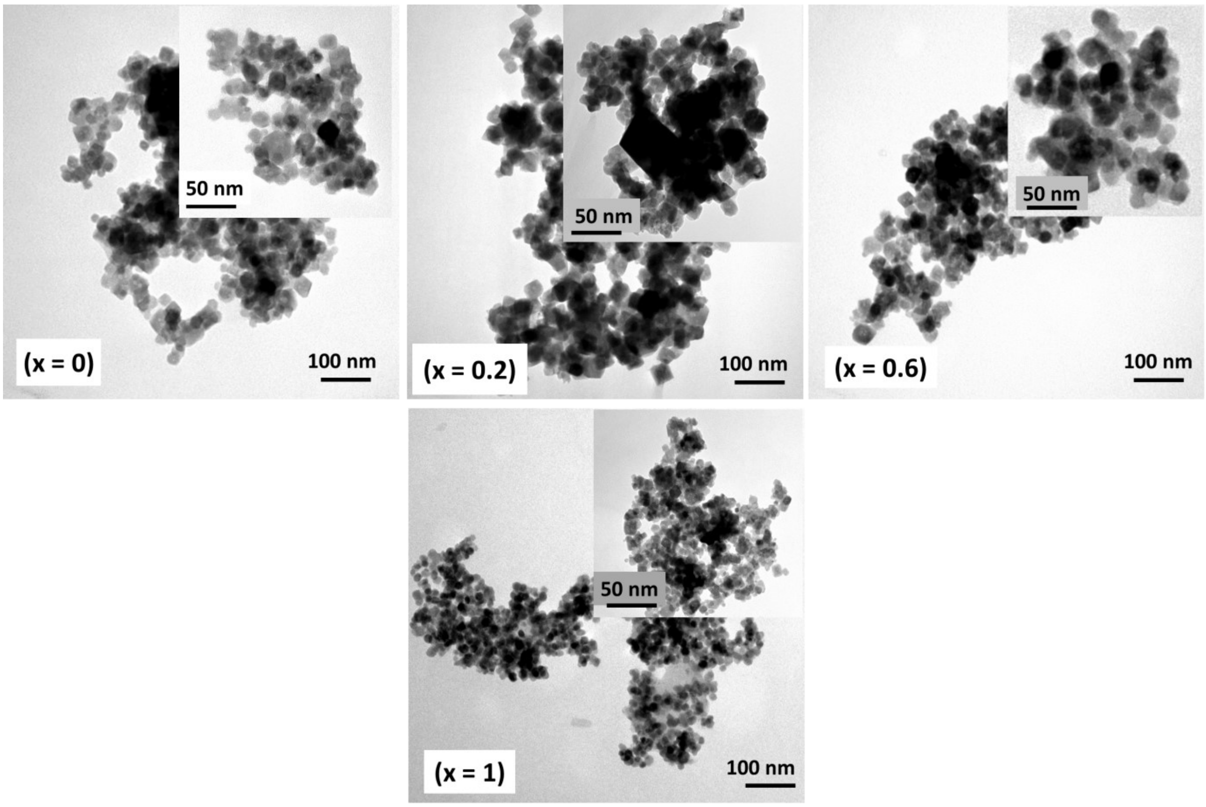

Fig. S4. TEM images of $Ni_{1-x}Co_xFe_2O_4$ (0, 0.2, 0.6 and 1) nanoparticles.

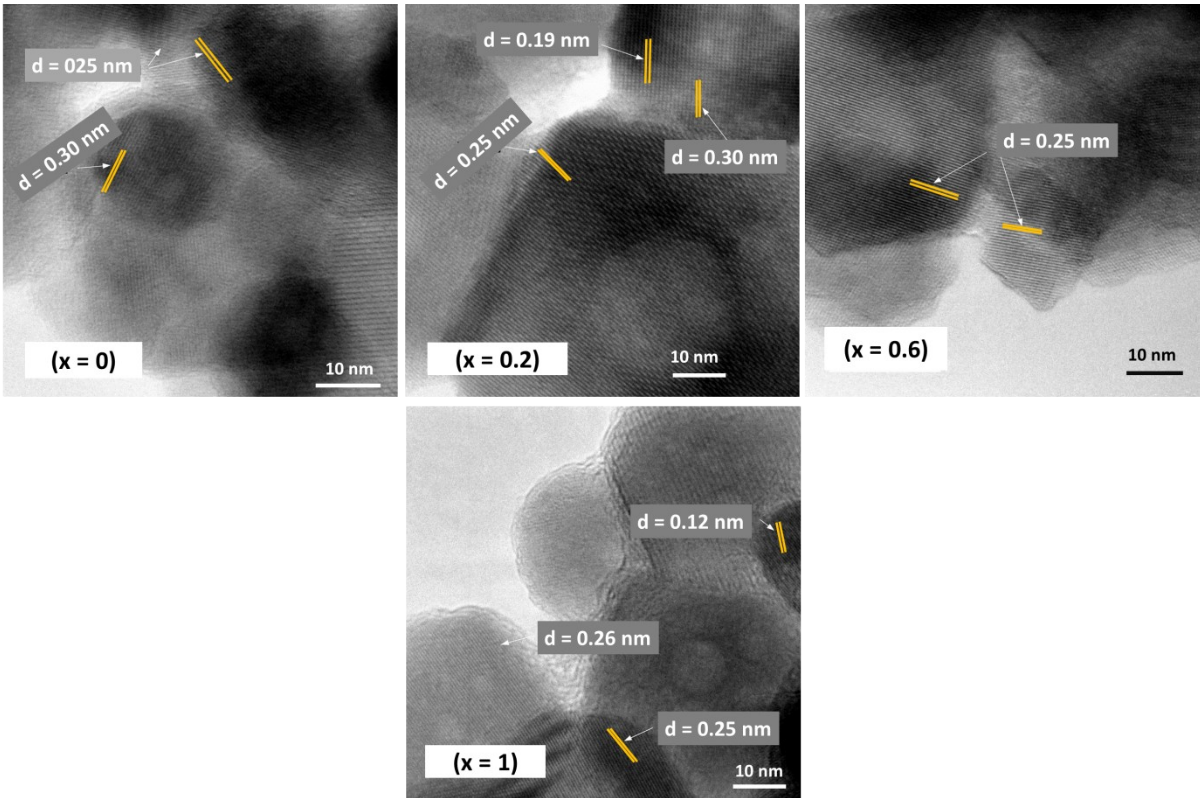

Fig. S5. HRTEM images of $Ni_{1-x}Co_xFe_2O_4$ (0, 0.2, 0.6 and 1) nanoparticles.

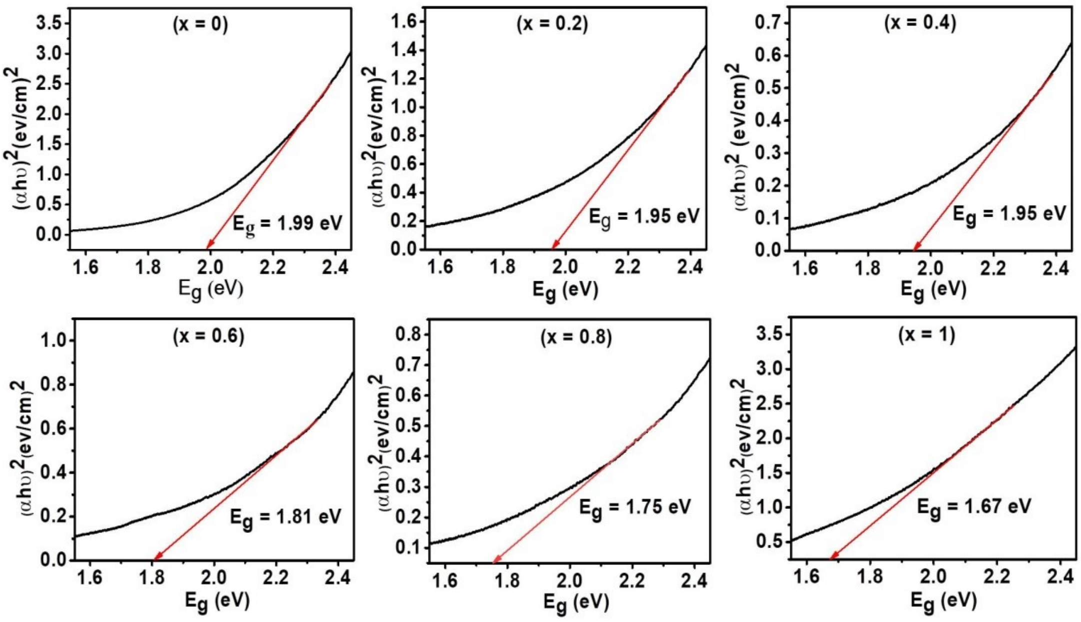

Fig. S6. Tauc plots of $(\alpha h\upsilon)^2$ versus energy for $Ni_{1-x}Co_xFe_2O_4$ ($0 \leq x \leq 1$) solid solutions.

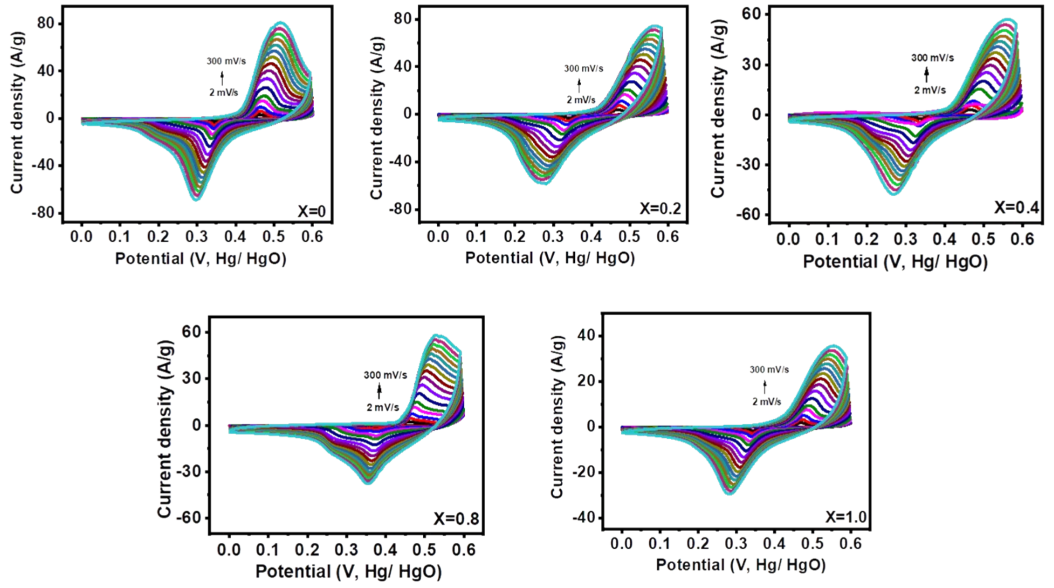

Fig. S7. CV curves of the $Ni_{1-x}Co_xFe_2O_4$ ($0 \leq x \leq 1$) samples at various scan rates.

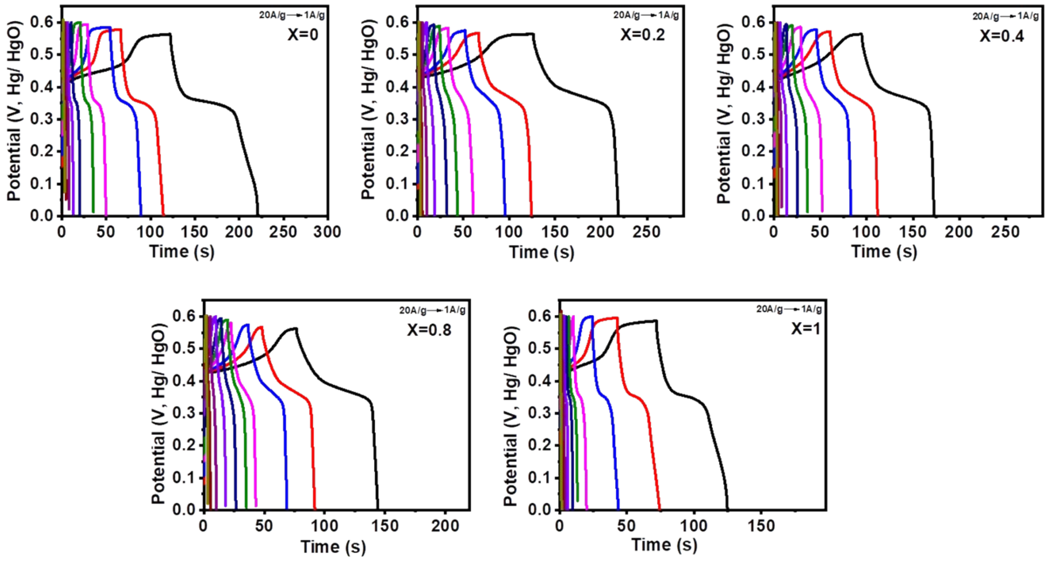

Fig. S8. Charge-discharge characteristics of $Ni_{1-x}Co_xFe_2O_4$ ($0 \leq x \leq 1$) samples at various current densities.

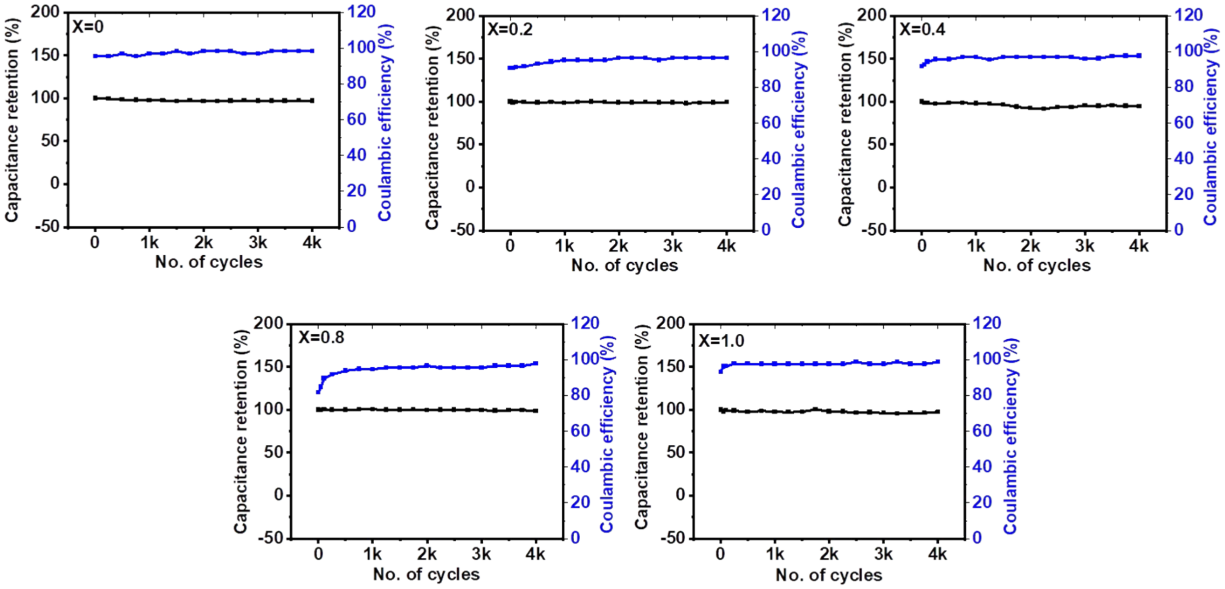

Fig. S9. Capacitance retention and columbic efficiency of various samples.

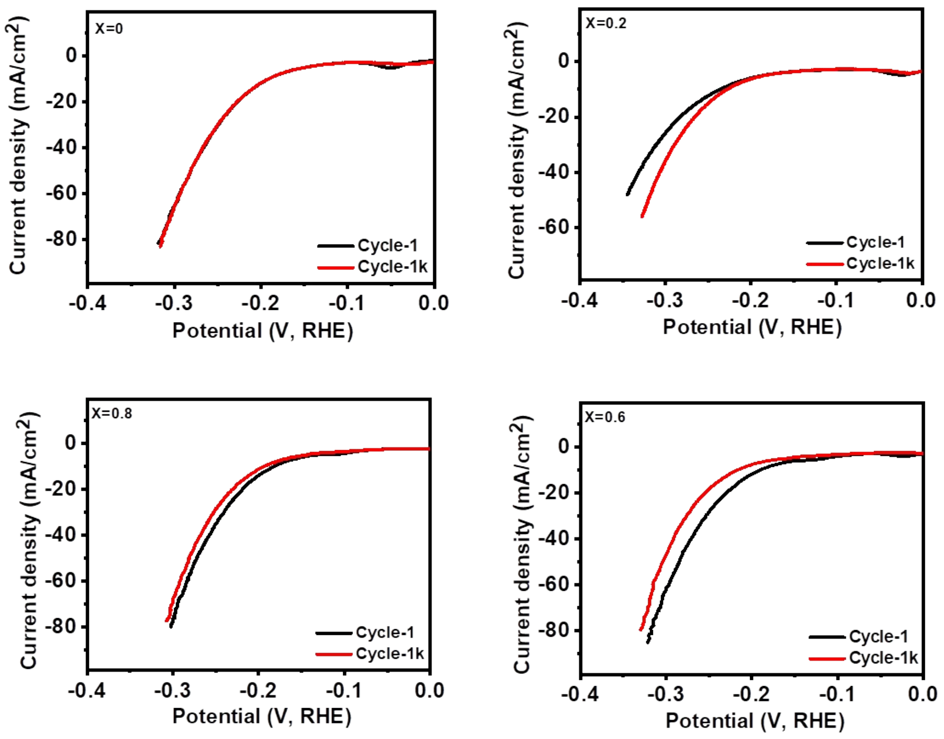

Fig. S10. HER polarization curves at various cycles for the various samples.

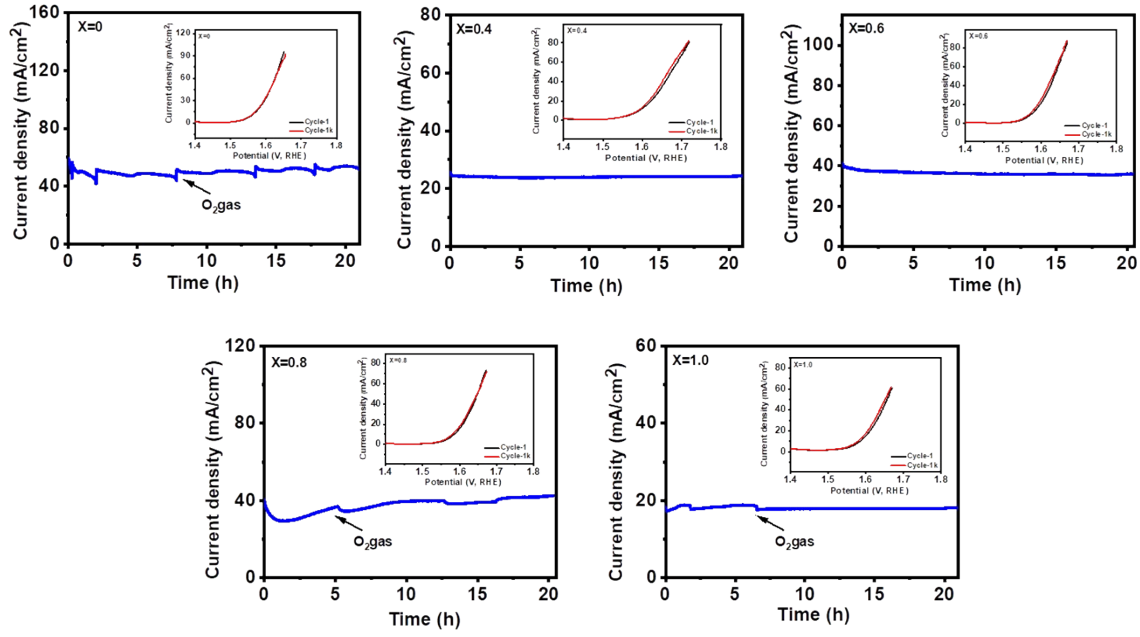

Fig. S11. Chronoamperometry characteristics of the various samples in 1M KOH (inset) OER polarization curves at various cycles for the various samples.

Table S1. Mass of precursors used in the synthesis of $Ni_{1-x}Co_xFe_2O_4$ ($0 \leq x \leq 1$) solid solutions.

**(x)**	**Target formula**	**Mass of Ni(acac)$_2$ (g)**	**Mass of Co(acac)$_3$ (g)**	Mass of Fe(acac)$_3$ (g)
0	$NiFe_2O_4$	0.100	-	0.275
0.2	$Ni_{0.8}Co_{0.2}Fe_2O_4$	0.080	0.028	0.275
0.4	$Ni_{0.6}Co_{0.4}Fe_2O_4$	0.059	0.055	0.275
0.6	$Ni_{0.4}Co_{0.6}Fe_2O_4$	0.039	0.083	0.275
0.8	$Ni_{0.2}Co_{0.8}Fe_2O_4$	0.019	0.111	0.275
1.0	$CoFe_2O_4$	-	0.100	0.198

Table S2. Comparison of the specific capacitance of $Ni_{0.4}Co_{0.6}Fe_2O_4$ electrode with other metal oxide-based electrodes.

**Electrode material**	**Synthesis route**	**Specific capacitance (F/g)**	**Current density (A/g)**	**Reference**
$Cu_{0.5}Co_{0.5}Fe_2O_4$	Sol-gel	76.9	1	[1]
$MnFe_2O_4$	Co-precipitation	173	1	[2]
$MgFe_2O_4$	Sol-gel	61	0.5	[3]
$Ni_{0.5}Co_{0.5}Fe_2O_4$	Sol-gel	50	1	[1]
Fe_3O_4	Solvothermal	97	3	[4]
$NiCo_2O_4$	Sol-gel	217	1	[5]
$Ni_{0.5}Cu_{0.5}Fe_2O_4$	Sol-gel	44	1	[1]
$MnFe_2O_4$ /graphene	Solvothermal	120	0.1	[6]
$CoMnFeO_4$	Sol-gel	150	1	[7]
$CdMn_2O_4$	Electrospinning	210	1	[8]
$Ni_{0\cdot25}Mg_{0\cdot75}Fe_2O_4$	Hydrothermal	133.95	0.5	[9]
$MgCr_2O_4$	Sol-gel	21	0.5	[10]
**$Ni_{0.4}Co_{0.6}Fe_2O_4$**	**Solventless**	**237**	**1**	**This study**

Table S3. Comparison of HER performance of the synthesized $Ni_{0.6}Co_{0.4}Fe_2O_4$ and $CoFe_2O_4$ with other reported Ni/Co-based electrocatalysts in alkaline electrolyte.

Catalyst	Synthetic method	η_{10} (mV in 1 M KOH	Tafel slope (mV/dec)	Reference
NiFe Sponges	Polyol-assisted chemical synthesis	190	82	[11]
NiCoP NW/CFP	Hydrothermal	170	73.0	[12]
$Ni_{1.5}Co_{1.5}S_4$NW/CFP	followed by sulfuration and phosphorization	237	112.9	[12]
$CoFe_2O_4$/SWNTs	Sonochemical	263	46	[13]
Ni-$MoSe_2$	Hydrothermal	206	81	[14]
NiMnP	Colloidal	490	238	[15]
$FeSe_2/CoFe_2O_4$	Hydrothermal	231	88.76	[16]
CoCuZn/C	Electrodeposition	213	92	[17]
$NiCo_2S_4$/Ni foam	hydrothermal	210	-	[18]
$CoSe_2/MoSe_2$	Solvothermal	218	76	[19]
$NiCo_2O_4$/NiCoP	Solvothermal followed by phosphorization	198	91	[20]
**$CoFe_2O_4$**	**Solventless**	**169**	**113**	**This study**
**$Ni_{0.6}Co_{0.4}Fe_2O_4$**	**Solventless**	**168**	**120**	**This study**

Table S4. Comparison of OER performance of the synthesized $Ni_{0.8}Co_{0.2}Fe_2O_4$ with other reported Ni/Co-based electrocatalysts in alkaline electrolyte.

Catalyst	Synthetic method	η_{10} (mV) in 1 M KOH	Tafel slope (mV/dec)	Reference
NiS/Bi_2WO_6	Hydrothermal	527	238	[21]
$Ni_xCo_{3-x}O_4$/NF	Hydrothermal	320	38	[22]
$Ni_xCo_{3-x}O_4$ nonowires	Hydrothermal	337	75	[23]
Co/Fe-MOFs	Solvothermal	410	101	[24]
$MnO_2/NiCo_2O_4$/NF	Hydrothermal	340	139	[25]

$NiCo_2O_4$/NF	Solvothermal	465	137	[26]
NiCoP/C nanoboxes	MOF	330	96	[27]
Mn-Co oxyphosphide	Thermal oxidation and phosphidation	370	66	[28]
$CuCo_2S_4$	Colloidal	395	115	[29]
Mn-Co oxide	Thermal oxidation	420	60	[28]
$NiCo_2O_4$	Hydrothermal	346	94	[30]
$CoNi_{0.2}Fe_{0.05}$-Z-H-P	MOF and phosphidation	329	48.2	[31]
CoS	Electrodeposition	372	86.6	[32]
CoMnP nanoparticles	Solvothermal	330	61	[33]
CoP-PBSCF	*In-situ* exsolution	340	81.5	[34]
$Co_2Mo_3O_8$@NC	*In situ* pyrolysis	331	87.5	[35]
$NiCo_2O_4$	Hydrothermal	500	119	[25]
**$Ni_{0.8}Co_{0.2}Fe_2O_4$**	**Solventless**	**320**	**79**	**This study**

References

1. *Bhujun, B.; Tan, M. T.; Shanmugam, A. S. Results in Physics 2017, 7, 345-353.*
2. *Vignesh, V.; Subramani, K.; Sathish, M.; Navamathavan, R. Colloids and Surfaces A: Physicochemical and Engineering Aspects 2018, 538, 668-677.*
3. *Maitra, S.; Mitra, R.; Nath, T. Current Applied Physics 2021, 27, 73-88.*
4. *Tipsawat, P.; Wongpratat, U.; Phumying, S.; Chanlek, N.; Chokprasombat, K.; Maensiri, S. Applied Surface Science 2018, 446, 287-292.*
5. *Wu, Y. Q.; Chen, X. Y.; Ji, P. T.; Zhou, Q. Q. Electrochimica Acta 2011, 56, (22), 7517-7522.*
6. *Cai, W.; Lai, T.; Dai, W.; Ye, J. Journal of Power Sources 2014, 255, 170-178.*
7. *Ghadimi, L. S.; Arsalani, N.; Ahadzadeh, I.; Hajalilou, A.; Abouzari-Lotf, E. Applied Surface Science 2019, 494, 440-451.*
8. *Bhagwan, J.; Sahoo, A.; Yadav, K.; Sharma, Y. Journal of Alloys and Compounds 2017, 703, 86-95.*
9. *Wongpratat, U.; Tipsawat, P.; Khajonrit, J.; Swatsitang, E.; Maensiri, S. Journal of Alloys and Compounds 2020, 831, 154718.*
10. *Maitra, S.; Mitra, R.; Nath, T. Journal of Alloys and Compounds 2021, 858, 157679.*
11. *Thoufeeq, S.; Rastogi, P. K.; Thomas, S.; Shravani, A.; Narayanan, T. N.; Anantharaman, M. ChemistrySelect 2020, 5, (4), 1385-1395.*
12. *Dai, Z.; Geng, H.; Wang, J.; Luo, Y.; Li, B.; Zong, Y.; Yang, J.; Guo, Y.; Zheng, Y.; Wang, X. ACS nano 2017, 11, (11), 11031-11040.*
13. *Ding, Y.; Zhao, J.; Zhang, W.; Zhang, J.; Chen, X.; Yang, F.; Zhang, X. ACS Applied Energy Materials 2018, 2, (2), 1026-1032.*

14. Zhao, G.; Wang, X.; Wang, S.; Rui, K.; Chen, Y.; Yu, H.; Ma, J.; Dou, S. X.; Sun, W. Chemistry–An Asian Journal 2019, 14, (2), 301-306.
15. Man, H.-W.; Tsang, C.-S.; Li, M. M.-J.; Mo, J.; Huang, B.; Lee, L. Y. S.; Leung, Y.-c.; Wong, K.-Y.; Tsang, S. C. E. Applied Catalysis B: Environmental 2019, 242, 186-193.
16. Zhang, H.; Nengzi, L.-c.; Li, B.; Cheng, Q.; Gou, J.; Cheng, X. Renewable Energy 2020, 155, 717-724.
17. Döner, A. international journal of hydrogen energy 2018, 43, (51), 22797-22806.
18. Sivanantham, A.; Ganesan, P.; Shanmugam, S. Advanced Functional Materials 2016, 26, (26), 4661-4672.
19. Zhao, G.; Li, P.; Rui, K.; Chen, Y.; Dou, S. X.; Sun, W. Chemistry–A European Journal 2018, 24, (43), 11158-11165.
20. Jin, W.; Chen, J.; Wu, H.; Zang, N.; Li, Q.; Cai, W.; Wu, Z. Catalysis Science & Technology 2020, 10, (16), 5559-5565.
21. Li, J.; Xu, X.; Zhang, B.; Hou, W.; Lv, S.; Shi, Y. Applied Surface Science 2020, 526, 146718.
22. Bao, J.; Liu, W.; Xie, J.; Xu, L.; Guan, M.; Lei, F.; Zhao, Y.; Huang, Y.; Xia, J.; Li, H. Chemistry–An Asian Journal 2019, 14, (3), 480-485.
23. Yan, X.; Li, K.; Lyu, L.; Song, F.; He, J.; Niu, D.; Liu, L.; Hu, X.; Chen, X. ACS applied materials & interfaces 2016, 8, (5), 3208-3214.
24. Iqbal, B.; Saleem, M.; Arshad, S. N.; Rashid, J.; Hussain, N.; Zaheer, M. Chemistry–A European Journal 2019, 25, (44), 10490-10498.
25. Yan, K.-L.; Shang, X.; Gao, W.-K.; Dong, B.; Li, X.; Chi, J.-Q.; Liu, Y.-R.; Chai, Y.-M.; Liu, C.-G. Journal of Alloys and Compounds 2017, 719, 314-321.
26. Yin, X.; Sun, G.; Wang, L.; Bai, L.; Su, L.; Wang, Y.; Du, Q.; Shao, G. International Journal of Hydrogen Energy 2017, 42, (40), 25267-25276.
27. He, P.; Yu, X. Y.; Lou, X. W. Angewandte Chemie International Edition 2017, 56, (14), 3897-3900.
28. Guan, B. Y.; Yu, L.; Lou, X. W. Angewandte Chemie International Edition 2017, 56, (9), 2386-2389.
29. Wiltrout, A. M.; Read, C. G.; Spencer, E. M.; Schaak, R. E. Inorganic chemistry 2016, 55, (1), 221-226.
30. Jeghan, S. M. N.; Lee, G. Nanotechnology 2020, 31, (29), 295405.
31. Wang, M.; Dong, C. L.; Huang, Y. C.; Li, Y.; Shen, S. Small 2018, 14, (35), 1801756.
32. Xie, H.; Geng, Q.; Liu, X.; Mao, J. Frontiers of Chemical Science and Engineering 2021, 1-8.
33. Li, D.; Baydoun, H.; Verani, C. N.; Brock, S. L. Journal of the American Chemical Society 2016, 138, (12), 4006-4009.
34. Zhang, Y.-Q.; Tao, H.-B.; Chen, Z.; Li, M.; Sun, Y.-F.; Hua, B.; Luo, J.-L. Journal of Materials Chemistry A 2019, 7, (46), 26607-26617.
35. Ouyang, T.; Wang, X. T.; Mai, X. Q.; Chen, A. N.; Tang, Z. Y.; Liu, Z. Q. Angewandte Chemie International Edition 2020, 59, (29), 11948-11957.
